# Association of diabetes risk reduction diet with renal cancer risk in 101,755 participants: a prospective study

**DOI:** 10.1186/s12967-023-04555-z

**Published:** 2023-10-02

**Authors:** Ling Xiang, Yi Xiao, Zhiquan Xu, Haoyun Luo, Xiaorui Ren, Qi Wei, Zhiyong Zhu, Yahui Jiang, Yunhao Tang, Hongmei He, Zhihang Zhou, Haitao Gu, Yaxu Wang, Linglong Peng

**Affiliations:** 1 Department of Clinical Nutrition, The Second Affiliated Hospital of Chongqing Medical University, Chongqing, China; 2 Department of Gastrointestinal Surgery, The Second Affiliated Hospital of Chongqing Medical University, No. 288 Tianwen Avenue, Nan’an District, Chongqing, 400010 China; 3 Department of Gastroenterology, The Second Affiliated Hospital of Chongqing Medical University, Chongqing, China

**Keywords:** Diabetes risk reduction diet, Renal cancer, Cancer prevention, Epidemiology, Dietary pattern

## Abstract

**Background:**

There is little prospective evidence exists about whether adherence to a diabetes risk reduction diet (DRRD) is related to a significant reduction in renal cancer risk. We sought to clarify whether adherence to DRRD was associated with a reduced risk of renal cancer in a US population.

**Methods:**

A population-based cohort of 101,755 American adults was identified from the Prostate, Lung, Colorectal, and Ovarian Cancer Screening Trial. A DRRD score was calculated to assess adherence to this dietary pattern, where increased scores indicated greater adherence. The relationship between DRRD score and risk of renal cancer was assessed based on the hazard ratios (HRs) and 95% confidence intervals (CIs), which were both calculated using Cox regression. Non-linear association was determined through restricted cubic spline regression. Potential effect modifiers were identified through subgroup analyses.

**Results:**

Over a mean follow-up of 8.8 years, 446 renal cancers were detected. In this analysis, the fully adjusted model depicted a notable 29% reduction in the risk of renal cancer among individuals in the highest quartile of DRRD score in comparison with the lowest quartile individuals (HR_Q4 vs. Q1_: 0.71; 95% CI = 0.54, 0.94; *P*_trend_ = 0.008). This association remained consistent across a series of sensitivity analyses. A non-linear inverse dose–response association between renal cancer risk with DRRD score was observed (*P*_nonlinearity_ = 0.026). Subgroup analyses showed that this favorable link was more prominent in participants with low Healthy Eating Index-2015 (*P*_interaction_ = 0.015). Regarding the individual components of DRRD, a decrease in the risk of renal cancer was linked to increased intake of cereal fiber and whole fruit, and lower sugar-sweetened beverage consumption (all *P*_trend_ < 0.05).

**Conclusions:**

Our findings indicate that individuals adhering to DRRD are associated with a reduction in the risk of renal cancer.

**Supplementary Information:**

The online version contains supplementary material available at 10.1186/s12967-023-04555-z.

## Introduction

Renal cancer, with an annual incidence rate of over 330,000 cases, is regarded as the 13th most prevalent cancer globally [1]. There is significant geographic variation in the incidence of renal cancer. Europe and North America are the regions with the highest burden of renal cancer, and the low-risk countries are mainly located in Asia and Africa with an incidence of less than 2/100,000 [2]. This regional difference may be attributed to the increased prevalence of diabetes and obesity in western countries, in which western dietary patterns may play an important role [3, 4]. Several studies have linked diabetes to the incidence of renal cancer and have identified it as an independent risk factor [5]. The underlying mechanisms primarily involve insulin resistance, pro-inflammatory effect, and compensatory excess insulin production, which exhibit overlapping mechanisms with cancer [6–8]. Recently, a specific dietary pattern was developed to prevent diabetes, namely a diabetes risk reduction diet (DRRD). This dietary pattern emphasizes the higher intakes of grain fiber, nuts, coffee, polyunsaturated fatty acids, and whole fruit, and a lesser intake of saturated and trans fatty acids, red and processed meats, sugar-sweetened beverages, and lower values of glycemic index [9]. Along with being linked to a decreased risk of developing diabetes, adherence to DRRD has been linked to a lower risk of pancreatic cancer [10], breast cancer [11], endometrial cancer [12], and lung cancer [13]. However, epidemiologic evidence regarding the association between DRRD and the risk of renal cancer is currently lacking in literature. Therefore, a large-scale prospective study was performed to investigate the impact of adherence to the DRRD on the mitigation of renal cancer risk.

## Methods

### Study design

The population under study was retrieved from the Prostate, Lung, Colorectal, and Ovarian (PLCO) Cancer Screening Trial. Funded and designed by the United States National Cancer Institute (NCI), the PLCO is a randomized large-scale clinical trial that explores the effectiveness of screening methods in reducing fatality from various cancers in both men and women, such as prostate, lung, colorectal, and ovarian cancers [14]. The PLCO trial spanned from 1993 to 2001 across ten medical centers in the United States involving a total of 154,887 individuals (aged 55 to 74 years) being included in the trial. Following the provision of informed consent, the participants, per the PLCO trial design, were assigned randomly to equal proportions of control or intervention groups. The control group was provided the usual care, while the intervention group received screening tests. All the participating individuals were instructed to complete a self-reported questionnaire on lifestyle and recorded cancer incidence up until 2009. Specific questionnaire information included the Baseline Questionnaire (BQ) and the Dietary History Questionnaire (DHQ) [14]. The BQ was primarily employed to retrieve the baseline risk factors of participants at enrollment and the diagnosis information of cancers. The DHQ collected dietary information from participants using a 124-item Food Frequency Questionnaire (FFQ) to examine foods or nutrients intake over the past year. Previous studies have indicated the effectiveness of the DHQ in assessing diet and nutrient intake [15, 16]. Further comprehensive data about the PLCO trial can be found in the literature [14, 17].

**Fig. 1:**
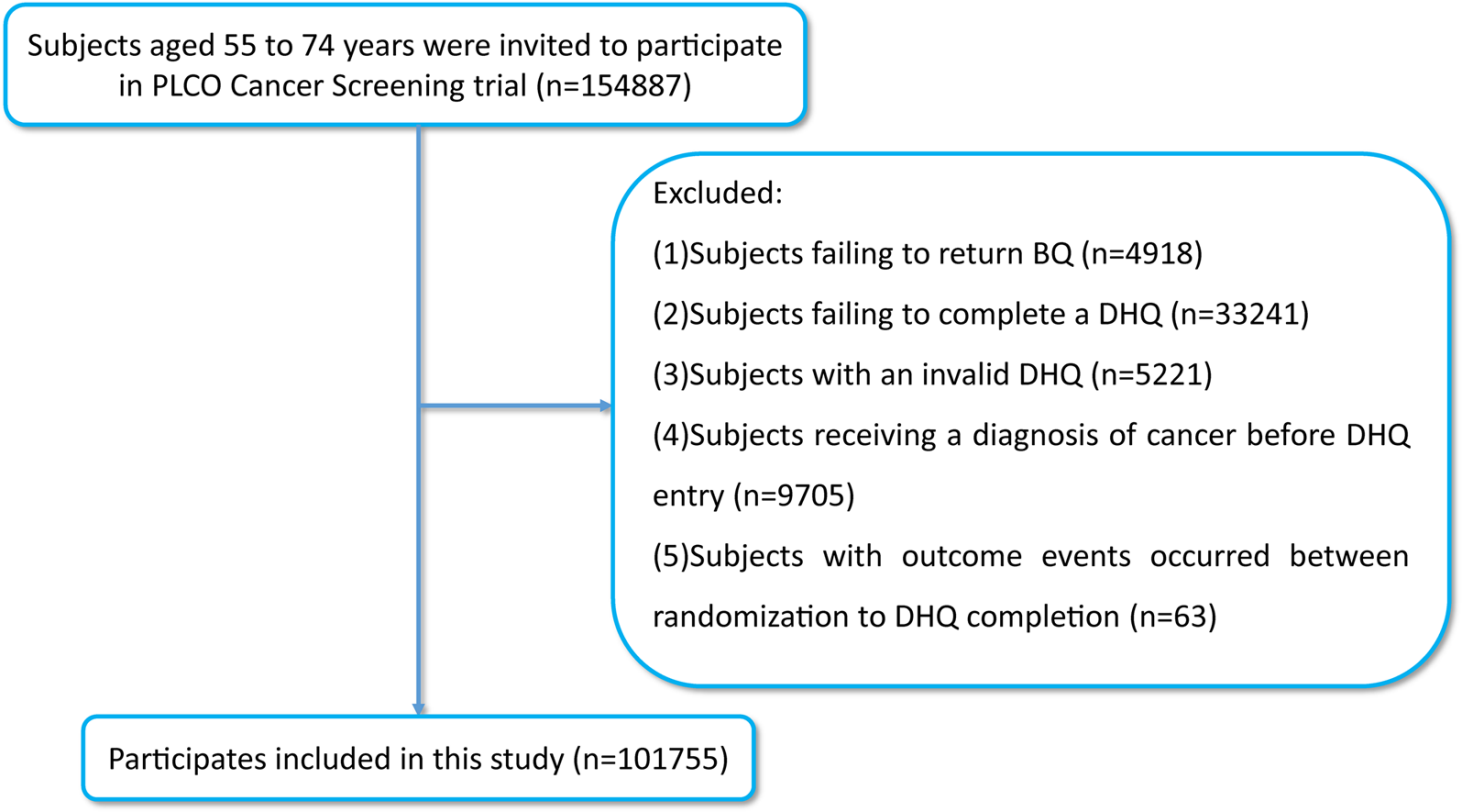
The flow chart of identifying eligible subjects. PLCO, Prostate, Lung, Colorectal, and Ovarian; BQ, baseline questionnaire; DHQ, diet history questionnaire

In this experiment, participants meeting the following criteria were excluded: (1) failing to return BQ (n = 4918); (2) failing to complete a DHQ (n = 33,241); (3) having an invalid DHQ (the DHQ had to meet certain requirements, including having a specific completion date, having a death date that was not prior to the completion date, having fewer than 8 missing frequency responses, and having an energy intake without extremes (i.e., not in the top 1% or bottom 1% of intake) (n = 5221); (4) being diagnosed with cancer prior to DHQ entry (n = 9705); and (5) developing outcome events such as renal cancer, died, or were lost to follow-up between randomization and completion of the DHQ were excluded (n = 63). Ultimately, 101,755 participants in total met the eligibility criteria **(**Fig. 1**)**. Approval for this research was granted by the NCI (Project ID: PLCO-1063).

### DRRD score calculation

Based on the latest published research [18, 19], the DRRD score was quantified to assess the adherence of participants to a type 2 diabetes prevention diet. The individuals were classified into five strata per the quintile of their dietary intakes for each of the nine DRRD components and given points based on their strata (Additional file 1: Table S1). Participants with the highest intake of favorable components (highest stratum), such as coffee, cereal fiber, whole fruits, and polyunsaturated to saturated fatty acids ratio, and nuts, receive 5 points, whereas those with the lowest intake (lowest stratum) receive 1 point. In addition, the trend was the opposite for the “unfavorable” DRRD components, including glycemic index, trans fatty acids, sugar-sweetened beverages, and red and processed meats. The scores for the nine components were then summed, resulting in a total DRRD score ranging from 9 to 45 for each individual. A higher score indicates greater adherence to the DRRD.

This research utilized the aforementioned DHQ to extract the nutrient variables used for the purpose of calculating the DRRD score. The cereal fiber refers to insoluble fiber collected in DHQ, and sugar-sweetened beverages are the sum of fruit drinks and soft drinks assessed by DHQ. The glycemic index was derived using the DHQ data according to the method described in previous literature [20]. It is reliable and reproducible to assess nutrition-related indicators via the DHQ [21].

### Covariates data collection

The personal baseline information of participants was available in the completed self-administered BQ. In this study, the following factors: level of education, race/ethnicity, age, marital status, sex, body mass index (BMI), weight change, arm (intervention or control), pack-years of smoking, smoking status, ibuprofen use, family history of renal cancer, history of diabetes and hypertension were included. BMI was derived as weight (kg) divided by height squared (m^2^) at baseline. Weight change was termed as the baseline weight of the participant minus weight at age 20 (pounds). DHQ was utilized to retrieve dietary data, such as alcohol consumption, total energy intake from diet, Healthy Eating Index (HEI)-2015 score, and individual dietary components of DRRD. It is worth noting that HEI-2015 was utilized to assess personal diet quality, and the specific calculation method can be checked in prior research [22].

### Renal cancer ascertainment

The PLCO trial used a method of identifying cases of renal cancer based on reports extracted from the annual study update form, which included self-reports, reports from family members, and death certificates, among other sources. After the identification of potential cases, the medical records were reviewed to confirm the diagnoses using ICD-O-2 codes, and data were extracted using standardized tables. The endpoint of this study was defined as malignant neoplasm of renal parenchyma and renal pelvis (C649 and C659).

### Statistical analysis

In this study, all missing data were less than 5%. Hence, the missing data of categorical covariates, which included educational degree, marital status, family history of renal cancer, ibuprofen use, and smoking status as well as a history of hypertension, and diabetes were imputed using the modal value. The missing data of continuous covariates, including those such as pack-years of smoking, BMI, and weight change, were imputed with median value. The detailed imputation information was depicted in tabular form (Additional file 1: Table S2).

**Fig. 2:**
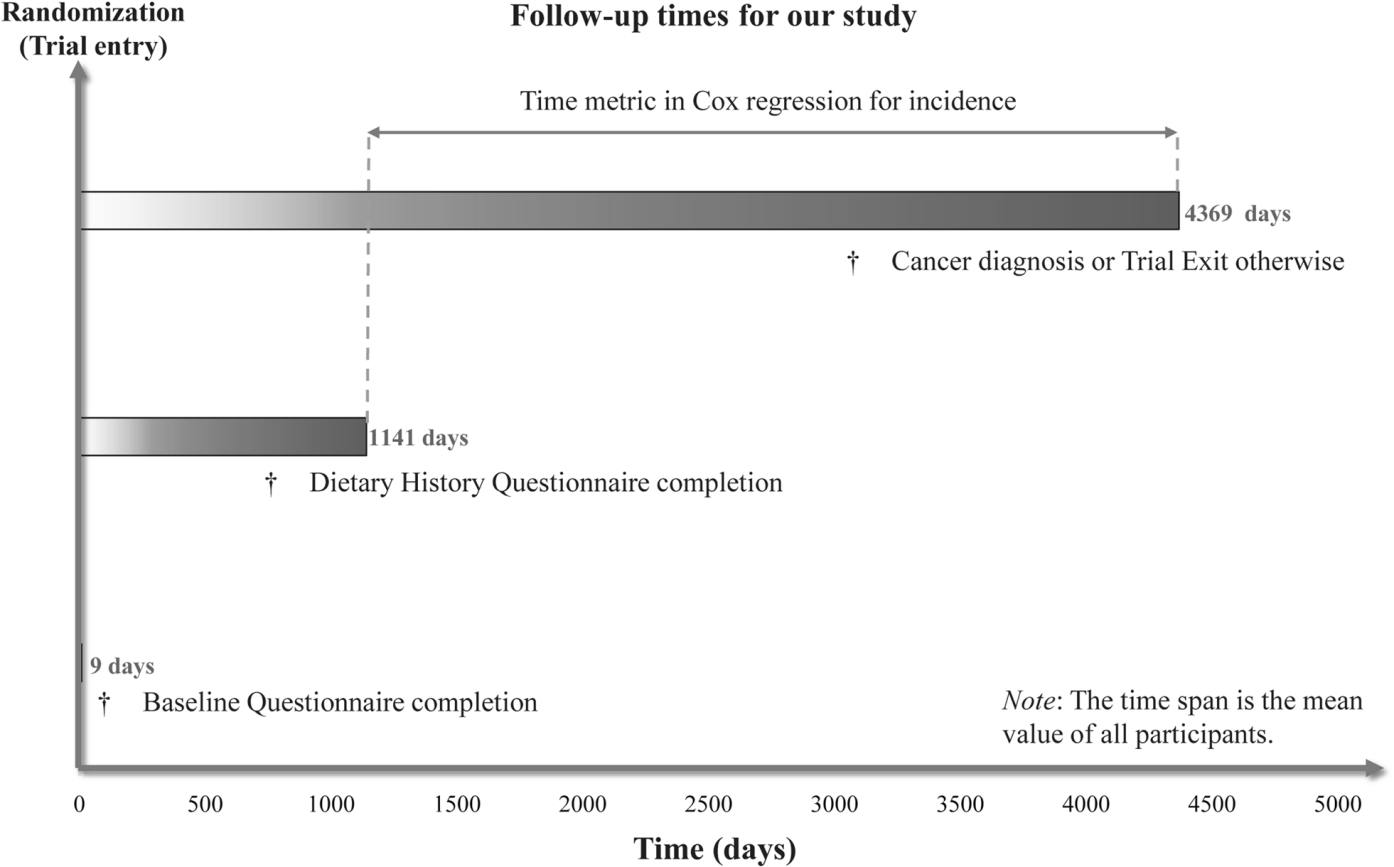
The timeline and follow-up scheme of our study

A Cox proportional hazards regression model was employed to determine the 95% confidence intervals (CIs) and hazard ratios (HRs) for assessing the correlation between DRRD score and renal cancer incidence, with the follow-up period as the time metric. In particular, this research defined the follow-up period as the time from the completion of DHQ to the incidence of renal cancer, fatality, the loss of follow-up, or the end of follow-up (i.e., December 31, 2009), whichever was the first to occur (Fig. 2). In the Cox regression model, the DRRD score was classified into quartiles, with the reference group defined as the lowest quartile. Based on the follow-up period, the person-years of each quartile were quantified. Schoenfeld residuals approach was used to determine whether the DRRD score is a time-varying variable. The Cox regression model was utilized to conduct a trend test across the quartiles of DRRD scores for the risk assessment of renal cancer risk. This analysis involved assigning the median value of each quartile to all individuals within that quartile and treating it as a continuous variable. Afterward, covariates were screened and adjustments were made for confounders in multivariate regression models as per the review of the literature [23, 24] and clinical judgment. Adjustment of model 1 with race, sex, marital status, age, and education levels was done, and model 2 was further adjusted with smoking status, intake of alcohol, pack-years of smoking, BMI, trail arm, ibuprofen usage, family history of renal cancer, history of hypertension and diabetes, and energy intake from the diet. The study applied a restricted cubic spline model with three knots at the 10th, 50th, and 90th to evaluate the risk of renal cancer across the full spectrum of the DRRD score [25].

The study conducted several pre-selected subgroup analyses to determine whether the relationship between DRRD score and renal cancer incidence was influenced by certain factors. These included sex (male vs. female), age (≤ 65 vs. > 65 years old), BMI (≤ 30 vs. > 30 kg/m2), smoking status (non-smokers vs. current/former smoker), pack-years of smoking (≤ medium vs. > medium), family history of renal cancer (no vs. yes/possible), history of hypertension (no vs. yes), history of diabetes (no vs. yes) and HEI-2015 (≤ medium vs. > medium). The P value for interaction was calculated to indicate the intrinsic statistical interaction in these subgroup analyses.

To ensure the reliability of the obtained data, multiple sensitivity analyses were conducted. These measures included (1) exclusion of individuals with a family history of renal cancer due to their higher predisposition to develop renal malignancy; (2) exclusion of individuals with a diabetic history as they may have a greater tendency to adhere to a diabetes risk reduction diet; (3) exclusion of 32 cases with renal pelvis cancer, to clarify the association of DRRD score with the renal parenchyma cancer risk; (4) exclusion of observed cases within the initial years of follow-up (first two and three years) to eliminate the issue of reverse causality; (5) additional adjustment in model 2 for HEI-2015 to verify whether the determined correlation was influenced by the quality of the diet; and (6) adjustment for weight change in model 2 rather than BMI to examine the impact of body weight on the correlation.

The association of each dietary component of DRRD with renal cancer risk was assessed. Individual components of DRRD were categorized into quartiles, with the lowest quartile as the reference group. Subsequent analyses were similar to the above-mentioned methods for assessing the link of DRRD score with renal cancer risk. The descriptive statistics are expressed as mean ± standard deviation and number (percentage) for continuous and categorical variables. The statistical analyses were conducted utilizing the R software (version 4.0.5). A two-tailed *p*-value less than 0.05 was considered significant.

## Results

### Participant baseline features

In this research, the number of participants reached 101,755 as per the above-mentioned exclusion criteria (Fig. 1). The mean (standard deviation) for DRRD score, age, and follow-up time of participants were 26.8 (5.3) points, 65.5 (5.7) years, and 8.8 (1.9) years, respectively. The individuals were classified into quartiles by DRRD score [Quartile 1 (DRRD score, 9–23), n = 27,890; Quartile 2 (DRRD score, 24–27), n = 28,970; Quartile 3 (DRRD score, 28–30), n = 19,784; Quartile 4 (DRRD score, 31–45), n = 25,111]. Participants in higher quartiles indicated better adherence to a diabetes risk-reduction diet. Compared to the individuals in the lowest quartile group, those in the highest quartile were observed to have certain characteristics, such as were being older, female, non-smokers, and drinkers, as well as having higher education levels, alcohol consumption, and HEI-2015 score. Conversely, the likelihood of higher BMI, a history of diabetes or hypertension, and higher energy was low. More detailed information was also examined (Table 1).

**Table 1:** Baseline characteristics of study population according to overall diabetes risk reduction diet score

Characteristics	Overall	Quartiles of overall Diabetes Risk Reduction Diet score			
		Quartile 1 (9–23)	Quartile 2 (24–27)	Quartile 3 (28–30)	Quartile 4 (31–45)
Number of participants	101,755	27,890	28,970	19,784	25,111
Diabetes risk reduction diet score	26.8 ± 5.3	20.4 ± 2.4	25.5 ± 1.1	28.9 ± 0.8	33.8 ± 2.6
Age	65.5 ± 5.7	64.7 ± 5.6	65.5 ± 5.7	65.9 ± 5.8	66.2 ± 5.7
Sex					
Male	49,496 (48.6%)	16,282 (58.4%)	14,856 (51.3%)	8839 (44.7%)	9519 (37.9%)
Female	52,259 (51.4%)	11,608 (41.6%)	14,114 (48.7%)	10,945 (55.3%)	15,592 (62.1%)
Race					
White	94,066 (92.4%)	26,077 (93.5%)	26,890 (92.8%)	18,295 (92.5%)	22,804 (90.8%)
Non-white	7689 (7.6%)	1813 (6.5%)	2080 (7.2%)	1489 (7.5%)	2307 (9.2%)
Marital status					
Married or living as married	79,826 (78.4%)	22,143 (79.4%)	23,066 (79.6%)	15,509 (78.4%)	19,108 (76.1%)
No	21,929 (21.6%)	5747 (20.6%)	5904 (20.4%)	4275 (21.6%)	6003 (23.9%)
Education level					
College below	64,953 (63.8%)	19,955 (71.5%)	19,034 (65.7%)	12,053 (60.9%)	13,911 (55.4%)
College graduate	17,848 (17.5%)	4210 (15.1%)	4973 (17.2%)	3687 (18.6%)	4978 (19.8%)
Postgraduate	18,954 (18.6%)	3725 (13.4%)	4963 (17.1%)	4044 (20.4%)	6222 (24.8%)
Body mass index (kg/m^2^)	27.2 ± 4.8	28.2 ± 5.0	27.5 ± 4.7	26.9 ± 4.6	26.0 ± 4.4
Weight change^a^ (pounds)	33.0 ± 27.2	38.5 ± 28.8	34.6 ± 26.9	31.5 ± 26.4	26.4 ± 24.8
Arm					
Intervention	51,817 (50.9%)	14,077 (50.5%)	14,663 (50.6%)	10,063 (50.9%)	13,014 (51.8%)
Control	49,938 (49.1%)	13,813 (49.5%)	14,307 (49.4%)	9721 (49.1%)	12,097 (48.2%)
Smoking status					
Never	48,580 (47.7%)	12,409 (44.5%)	13,572 (46.8%)	9700 (49.0%)	12,899 (51.4%)
Current	9401 (9.2%)	3772 (13.5%)	2841 (9.8%)	1488 (7.5%)	1300 (5.2%)
Former	43,774 (43.0%)	11,709 (42.0%)	12,557 (43.3%)	8596 (43.4%)	10,912 (43.5%)
Smoking pack-years	17.7 ± 26.6	21.7 ± 30.0	18.4 ± 27.0	16.1 ± 25.0	13.5 ± 22.2
Drinking status					
No	27,757 (27.3%)	8654 (31.0%)	7780 (26.9%)	5006 (25.3%)	6317 (25.2%)
Yes	73,998 (72.7%)	19,236 (69.0%)	21,190 (73.1%)	14,778 (74.7%)	18,794 (74.8%)
Alcohol consumption (g/day)	9.5 ± 25.3	8.4 ± 23.5	9.7 ± 24.4	10.7 ± 28.1	9.7 ± 25.7
Ibuprofen use					
No	73,349 (72.1%)	19,784 (70.9%)	20,817 (71.9%)	14,243 (72.0%)	18,505 (73.7%)
Yes	28,406 (27.9%)	8106 (29.1%)	8153 (28.1%)	5541 (28.0%)	6606 (26.3%)
Family history of renal cancer					
No	97,569 (95.9%)	26,624 (95.5%)	27,766 (95.8%)	19,007 (96.1%)	24,172 (96.3%)
Yes	1543 (1.5%)	406 (1.5%)	423 (1.5%)	310 (1.6%)	404 (1.6%)
Possibly	2643 (2.6%)	860 (3.1%)	781 (2.7%)	467 (2.4%)	535 (2.1%)
History of diabetes					
No	94,949 (93.3%)	25,675 (92.1%)	26,957 (93.1%)	18,531 (93.7%)	23,786 (94.7%)
Yes	6806 (6.7%)	2215 (7.9%)	2013 (6.9%)	1253 (6.3%)	1325 (5.3%)
History of hypertension					
No	68,707 (67.5%)	18,002 (64.5%)	19,251 (66.5%)	13,558 (68.5%)	17,896 (71.3%)
Yes	33,048 (32.5%)	9888 (35.5%)	9719 (33.5%)	6226 (31.5%)	7215 (28.7%)
Energy intake from diet (kcal/day)	1738.6 ± 736.4	1797.0 ± 740.6	1739.3 ± 782.4	1715.3 ± 763.7	1691.5 ± 645.2
Healthy Eating Index-2015	66.5 ± 9.7	57.5 ± 7.9	65.2 ± 7.0	69.9 ± 6.5	75.5 ± 6.2
DRRD and other nutrients intakes					
Cereal fiber (g/day)	11.9 ± 5.7	9.3 ± 4.2	11.1 ± 5.2	12.5 ± 5.6	15.1 ± 6.2
Nuts (g/day)	6.7 ± 14.5	2.7 ± 5.7	4.9 ± 9.9	7.4 ± 14.8	12.7 ± 21.8
Coffee (g/day)	846.4 ± 794.5	730.2 ± 788.9	869.6 ± 802.6	892.7 ± 790.5	912.2 ± 780.6
Polyunsaturated/saturated fatty acids	0.8 ± 0.3	0.6 ± 0.2	0.7 ± 0.2	0.8 ± 0.2	0.9 ± 0.3
Whole fruit (Servings/day)	2.7 ± 2.0	1.7 ± 1.3	2.4 ± 1.7	3.0 ± 2.0	4.0 ± 2.4
Glycemic index of diet	53.6 ± 3.3	55.6 ± 3.0	53.9 ± 3.0	52.8 ± 2.9	51.4 ± 2.7
Trans fat acid (g/day)	4.0 ± 2.4	4.9 ± 2.5	4.2 ± 2.5	3.7 ± 2.2	2.9 ± 1.6
Sugar-sweetened beverage (g/day)	264.5 ± 433.3	449.6 ± 565.5	264.4 ± 407.3	191.5 ± 329.2	116.6 ± 254.4
Red and processed meat (g/day)	12.4 ± 15.3	19.5 ± 19.3	13.4 ± 15.0	9.8 ± 11.8	5.5 ± 7.6

Descriptive statistics are presented as (mean ± standard deviation) and number (percentage) for continuous and categorical. DRRD, diabetes risk reduction diet

^a^Weight change was defined as the participant's baseline weight minus weight at age 20

### Association between DRRD score and the incidence of renal cancer

During 899,337.5 person-years of follow-up, 446 renal cancer cases were observed, with an overall incidence rate of 0.496 cases per 1000 person-years. In the unadjusted model, in contrast with the lowest quartile of the DRRD score, the highest quartile individuals depicted a notably decreased incidence of renal cancer (HR_Q4 vs. Q1:_ 0.52; 95% CI 0.40, 0.68; *P* < 0.001 for trend) (Table 2). In the multivariate Cox regression model 1 and model 2 with adjustment for possible confounders, the DRRD score and the renal cancer incidence were depicted to have an inverse association [(model 1: HR _Quartile 4 vs. Quartile1_: 0.61; 95% CI 0.46, 0.80; *P* < 0.001 for trend) and (model 2: HR_Q4 vs. Q1_: 0.71; 95% CI 0.54, 0.94; *P* = 0.008 for trend)] (Table 2). It is important to note that this inverse association was similar when repeated analyses were executed in a population of 97,486 participants with the complete data, after excluding all missing data (HR_Q4 vs. Q1:_ 0.74; 95% CI 0.55, 0.98; *P* = 0.019 for trend) (Additional file 1: Table S3).

**Table 2:** Hazard ratios of the association of diabetes risk reduction diet score with the risk of renal cancer

Quartiles of DRRD score	Number of cases	Person-years	Incidence rate per 1000 person-years (95% confidence interval)	Hazard ratio (95% confidence interval)		
				Unadjusted	Model 1^a^	Model 2^b^
Quartile 1 (9–23)	162	243,233.1	0.67 (0.57, 0.78)	1.00 (reference)	1.00 (reference)	1.00 (reference)
Quartile 2 (24–27)	133	256,240.4	0.52 (0.44, 0.62)	0.78 (0.62, 0.98)	0.81 (0.64, 1.02)	0.85 (0.68, 1.08)
Quartile 3 (28–30)	73	175,613.0	0.42 (0.33, 0.52)	0.62 (0.47, 0.82)	0.68 (0.52, 0.90)	0.75 (0.57, 1.00)
Quartile 4 (31–45)	78	224,251.0	0.35 (0.28, 0.43)	0.52 (0.40, 0.68)	0.61 (0.46, 0.80)	0.71 (0.54, 0.94)
*P*_trend_				< 0.001	< 0.001	0.008

DRRD, diabetes risk reduction diet

^a^Model 1: Adjusted for age (years), sex (male, female), race (white, non-white), marital status (married or living as married, no), educational level (college below, college graduate, postgraduate)

^b^Model2: Adjusted for model 1 plus body mass index (kg/m2), smoking status (never, current, former), smoking pack-years (continuous), alcohol consumption (g/day), ibuprofen use (no, yes), arm (intervention, control), family history of renal cancer (no, yes), history of diabetes (no, yes), history of hypertension (no, yes) and energy intake from diet (kcal/day)

### Additional analyses

**Fig. 3:**
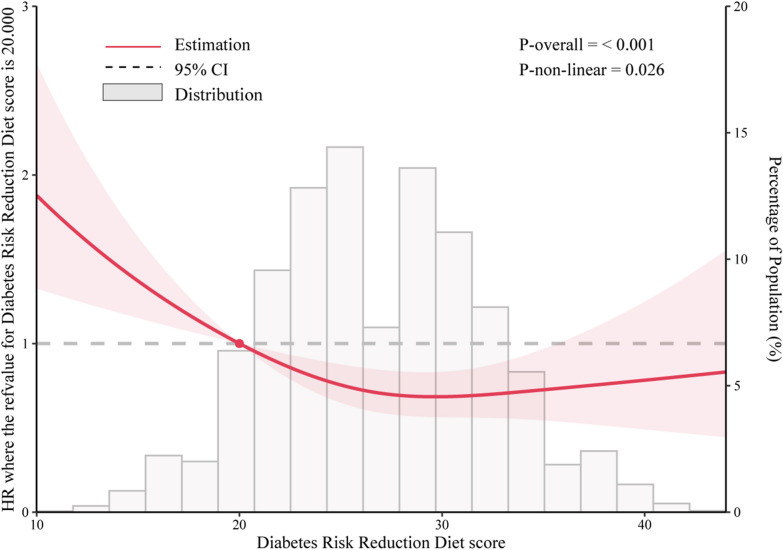
Nonlinear Dose–response analysis on the association of DRRD score with the risk of renal cancer. Hazard ratio was adjusted for age, sex, race, marital status, education levels, drinking status, alcohol consumption, smoking status, pack-years of smoking, body mass index, ibuprofen use, history of hypertension, history of diabetes, family history of renal cancer, and energy intake from diet (P = 0.026 for nonlinear nonlinearity)

A restricted cubic spline model was utilized to analyze the renal cancer risk across the full range of DRRD scores. The findings demonstrated that the association between DRRD score and renal cancer risk followed a nonlinear dose–response pattern and was an inverse association (*P* = 0.026 for nonlinearity) (Fig. 3). The analysis of the subgroups did not reveal any remarkable interaction between DRRD score and smoking status, age, consumption of alcohol, BMI, sex, family history of renal cancer, or history of diabetes and hypertension in relation to the occurrence of renal cancer (all *P* > 0.05 for interaction). The results, however, depicted that the inverse association between the DRRD score and renal cancer incidence was more prominent in individuals with lower Healthy Eating Index-2015 [≤ medium (67)] (HR_Q4 vs. Q1_: 0.29; 95% CI 0.11, 0.80; *P* = 0.015 for interaction) **(**Table 3**)**. In sensitivity analyses, the primary associations were still similar in the case of exclusion of cases with a family history of renal cancer (HR_Q4 vs. Q1_ = 0.69, 95% CI 0.52–0.92, *P* = 0.006 for trend) and exclusion of individuals with a history of diabetes (HR_Q4 vs. Q1_ = 0.74, 95% CI 0.55–0.99, *P* = 0.024 for trend) at baseline, exclusion of cases with renal pelvis cancer (HR_Q4 vs. Q1_ = 0.72, 95% CI 0.54–0.97, *P* = 0.020 for trend), exclusion of renal cancer cases during 2-year follow-up (HR_Q4 vs. Q1_ = 0.69, 95% CI 0.50–0.93, *P* = 0.005 for trend) or 3-year follow-up (HR_Q4 vs. Q1_ = 0.68, 95% CI 0.49–0.94, *P* = 0.008 for trend) baseline, further adjustment for Healthy Eating Index-2015 (HR_Q4 vs. Q1_ = 0.69, 95% CI 0.48–0.99, *P* = 0.035 for trend) and further adjustment for weight change (HR_Q4 vs. Q1_ = 0.71, 95% CI 0.53–0.93, *P* = 0.007 for trend) at baseline, implying the reliability of the inverse association between DRRD score and renal cancer risk (Table 4).

**Table 3:** Subgroup analyses on the association of diabetes risk reduction diet score with the risk of renal cancer

Subgroup variable	Number of participates	Number of cases	HR _Quartile 4 vs. Quartile 1_ (95% CI) ^a^	*P*_*-*interaction_
Age (years)				0.135
≤ 65	28,171	114	0.75 (0.39, 1.42)	
> 65	24,830	126	0.84 (0.48, 1.47)	
Sex				0.585
Male	25,801	156	0.74 (0.44, 1.27)	
Female	27,200	84	0.94 (0.47, 1.87)	
Body mass index (kg/m^2^)				0.455
≤ 30	40,972	161	0.93 (0.56, 1.53)	
> 30	12,029	79	0.55 (0.26, 1.18)	
Smoking status				0.289
Never	25,308	80	0.48 (0.24, 0.99)	
Current/former	27,693	160	1.05 (0.63, 1.75)	
Smoking pack-years				0.214
≤ Medium	26,872	85	0.45 (0.22, 0.91)	
> Medium	26,129	155	1.13 (0.67, 1.89)	
Family history of renal cancer				0.661
No	50,796	228	0.79 (0.52, 1.22)	
Yes/possibly	2205	12	1.34 (0.2, 8.81)	
History of hypertension				0.792
No	35,898	137	1.35 (0.77, 2.36)	
Yes	17,103	103	0.43 (0.23, 0.80)	
History of diabetes				0.394
No	49,461	219	0.87 (0.56, 1.34)	
Yes	3540	21	0.42 (0.10, 1.77)	
Healthy eating index-2015				0.015
≤ Medium	27,000	154	0.29 (0.11, 0.80)	
> Medium	26,001	86	1.12 (0.57, 2.20)	

HR, hazard ratio; CI, confidence interval

^a^HRs were adjusted for age (years), sex (male, female), race (white, non-white), marital status (married or living as married, no), educational level (college below, college graduate, postgraduate), body mass index (kg/m^2^), smoking status (never, current, former), smoking pack-years (continuous), alcohol consumption (g/day), ibuprofen use (no, yes), arm (intervention, control), family history of renal cancer (no, yes), history of diabetes (no, yes), history of hypertension (no, yes), Healthy Eating Index-2015 (continuous) and energy intake from diet (kcal/day)

**Table 4:** Sensitivity analyses on the association of diabetes risk reduction diet score with the risk of renal cancer

Categories	HR _Quartile 4 vs. Quartile 1_ (95% CI)^a^	P_-trend_
Excluded participants with a family history of renal cancer^b^	0.69 (0.52, 0.92)	0.006
Excluded participants with a history of diabetes^c^	0.74 (0.55, 0.99)	0.024
Exclude 32 cases with renal pelvis cancer	0.72 (0.54, 0.97)	0.020
Excluded cases observed within the first 2 years of follow-up	0.69 (0.50, 0.93)	0.005
Excluded cases observed within the first 3 years of follow-up	0.68 (0.49, 0.94)	0.008
Further adjusted for Healthy Eating Index-2015^d^	0.69 (0.48, 0.99)	0.035
Further adjusted for weight change^e^	0.71 (0.53, 0.93)	0.007

HR, hazard ratio; CI, confidence interval

^a^HRs were adjusted for age (years), sex (male, female), race (white, non-white), marital status (married or living as married, no), educational level (college below, college graduate, postgraduate), body mass index (kg/m^2^), smoking status (never, current, former), smoking pack-years (continuous), alcohol consumption (g/day), ibuprofen use (no, yes), arm (intervention, control), family history of renal cancer (no, yes), history of diabetes (no, yes), history of hypertension (no, yes) and energy intake from diet (kcal/day)

^b^HR was not adjusted for history of renal comorbidity

^c^HR was not adjusted for history of diabetes

^d^This covariate was treated as the continuous variable in multivariable Cox regression

^e^Weight change, defined as the participant's baseline weight minus weight at age 20, which was used as a proxy for BMI to be included in the analysis in the COX regression

### Dietary components of DRRD and the risk of renal cancer

Regarding the “favorable” DRRD components, less risk of renal cancer was depicted in individuals in the highest quartile of cereal fiber and whole fruit consumption in contrast to the lowest quartile [(cereal fiber: HR_Q4 vs. Q1_: 0.68; 95% CI 0.48, 0.97; *P* = 0.025 for trend) (Additional file 1: Table S4) and (whole fruit: HR_Q4 vs. Q1_: 0.69; 95% CI 0.52, 0.92; *P* = 0.008 for trend) (Additional file 1: Table S5)]. Among “unfavorable” DRRD components, individuals in the highest quartile of sugar-sweetened beverage intake had an elevated risk of renal cancer than those in the lowest quartile (HR_Q4 vs. Q1_: 1.46; 95% CI 1.10, 1.93; *P* = 0.004 for trend; Additional file 1: Table S6). There was a lack of any remarkable association between the risk of renal cancer and remaining DRRD components, including nuts (Additional file 1: Table S7), coffee (Additional file 1: Table S8), polyunsaturated/saturated fatty acids (Additional file 1: Table S9), glycemic index (Additional file 1: Table S10), trans fatty acids (Additional file 1: Table S11), and red meat and processed meat (Additional file 1: Table S12).

## Discussion

The PLCO Cancer Screening Trial provided the prospective data, which were utilized in this research to investigate the potential link between DRRD and renal cancer risk. Our result demonstrated that the DRRD score is associated with the incidence of renal cancer inversely, even after adjusting for established and suspected confounders. The restricted cubic spline model also presented a declining nonlinear trend of the incidence of renal cancer with increasing DRRD score, suggesting that the incidence of renal cancer is lower in individuals who adhered better to a diabetes risk reduction diet. Additionally, a series of sensitivity analyses were conducted, and the data depicted that the inverse association of the DRRD score with the renal cancer risk remained robust.

Previous studies have demonstrated that several single dietary or nutrient components are associated with renal cancer risk [26–28]. However, these studies have considerable limitations as they only consider the influence of single dietary component on cancer risk but ignore the possible antagonistic or synergistic effects between different dietary components. In the present study, a higher DRRD score, reflecting better adherence to the DRRD, is calculated by a series of dietary and nutrient factors, including a higher intake of coffee, nuts, cereal fiber, whole fruits, and higher values of the ratio of polyunsaturated to saturated fatty acids, and lower glycemic index, and lesser intake of trans fatty acids, sugar-sweetened beverage, and red and processed meats [18]. Given the intercorrelation and interaction between individual dietary components in influencing disease incidence, the application of certain dietary patterns to predict disease risk is appropriate and valuable [29, 30]. Currently, epidemiological studies have increasingly confirmed the potential correlation of DRRD patterns with reduced cancer risk, such as pancreatic, breast, endometrial, and lung cancers [10–13]. Although these results were consistent with these studies, the favorable impact of adherence to DRRD in preventing renal cancer has not been investigated in detail before, which may be a valuable contribution to the field of nutritional epidemiology of renal cancer.

The HEI-2015 is a diet quality indicator established by the United States Department of Agriculture, based on the Dietary Guidelines for Americans and the Food Pyramid. It consists of 13 food and nutrient components, including whole fruits, total fruits, whole grains, total vegetables, total protein foods, beans and greens, dairy, fatty acids, refined grains, sodium, seafood, plant proteins, added sugars, and saturated fats [31]. Notably, some overlapping dietary components were included in the dietary pattern of HEI-2015 and DRRD, such as whole fruits, fatty acids, and sugars. In this sensitivity analysis with further adjustment for HEI-2015, the inverse correlation between DRRD score and renal cancer risk did not change materially (HR_Q4 vs. Q1_: 0.69; 95% CI 0.48, 0.99; P = 0.035 for trend), suggesting that adherence to DRRD for reducing renal cancer risk is independent of diet quality. It has been shown that higher HEI-2015, indicating a better quality of diet, is associated with a lower incidence of cancers such as breast, oral, and pharyngeal cancers [31–33], but the evidence for the association of HEI-2015 and renal cancer risk was limited. In the subgroup analyses, the inverse association of DRRD scores with the risk of renal cancer was more prominent in individuals with a lower HEI-2015 [≤ medium (67)] (HR_Q4 vs. Q1_: 0.29; 95% CI 0.11, 0.80; *P* = 0.015 for interaction). This means that the potential benefit of adherence to DRRD in preventing renal cancer is greater for participants with poor diet quality.

An in-depth analysis to investigate the association of individual components of DRRD with renal cancer risk was conducted. The results suggested that renal cancer risk was negatively linked to cereal fiber and whole fruit intake, and positively linked to sugar-sweetened beverage intake. However, other components of DRRD did not depict any notable association with renal cancer risk. This means that cereal fiber, whole fruit, and sugar-sweetened beverage may be the main contributing components to the detected inverse association of DRRD with renal cancer incidence. Notably, in a meta-analysis designed to examine the relationship between dietary fiber and renal cancer risk, the researchers documented a strong correlation between the risk of renal cancer and vegetable and legume fiber intake, but not to the intake of cereal fiber [34]. The resulting data of this research provided valuable and additional evidence for the association of renal cancer risk with cereal fiber. Cereal fiber increases the chewing action and enhances the feeling of satiety after eating, which leads to weight loss [35] and thus plays a role in reducing cancer risk [36]. In addition, studies have demonstrated that whole fruit [37] and sugar-sweetened beverage consumption [38] are significantly associated with renal cancer risk, which is consistent with the results obtained. Accordingly, in specific dietary patterns (DRRD), the impact of individual components and potential synergies between different components on renal cancer risk deserves more attention.

The inverse association between DRRD and renal cancer risk in the study may be explained by the following mechanisms. It is known that diabetes is a metabolic disease characterized by chronic hyperglycemia, and the development of diabetes mainly includes the following three aspects: insulin resistance, pro-inflammatory effect, and compensatory excessive insulin production [39], which exhibits overlapping mechanisms with renal cancer [4]. First, insulin can directly or indirectly regulate the production of hepatic IGF-1 [40], and relevant clinical research has confirmed that elevated serum IGF-I levels are linked to a greater risk of renal cancer [6, 7] and that insulin resistance further enhances the above effects [8]. The role of chronic inflammation in promoting the occurrence and progression of cancer had also been affirmed [41]. Therefore, a possible explanation for the inverse correlation between DRRD scores and renal cancer risk is that individuals who adhere to the DRRD pattern are less likely to develop chronic inflammation, hyperinsulinemia, and insulin resistance, thus reducing the incidence of renal cancer. It is important to note that diabetes has been depicted as important concerning the etiology of renal cancer [42], and there is already established evidence of the association between DRRD and diabetes risk [9]. These findings imply that the association of the DRRD pattern with renal cancer risk observed in this study may be mediated by diabetes. However, after excluding patients with a history of diabetes in the sensitivity analysis, the initial correlation did not vary significantly (HR_Q4 vs. Q1_: 0.74; 95% CI 0.55, 0.99; P = 0.024 for trend), suggesting that the influence of this dietary pattern on renal cancer prevention is independent of the presence of diabetes. Further research is needed on the mechanism of the process to clarify these findings.

It should be emphasized that this study is the first to reveal the inverse association between DRRD score and renal cancer incidence in a large-scale population. Further sensitivity analyses confirmed the robustness of this inverse correlation. Additionally, subgroup analyses depicted that this association was more prominent in participants with lower HEI-2015 (≤ 67), suggesting that adherence to DRRD was more beneficial in decreasing the risk of renal cancer in those with lower diet quality. Furthermore, the role of each specific component of DRRD was examined concerning the reduction of the risk of developing renal cancer. In addition to determining that whole fruit and SSB are significantly related to renal cancer risk, the data also depicted a considerable effect of cereal fiber on reducing the risk of renal cancer, which is the first time that the relationship between cereal fiber and the risk of renal cancer was depicted. This may provide more accurate guidance for people to adhere to DRRD, that is, consuming higher whole fruit and cereal fiber and lower SSB in the diet pattern of DRRD may be more beneficial in reducing renal cancer risk.

Objectively, this study is limited in several aspects. First, the food consumption of DRRD was only examined once with DHQ instead of long-term cumulative averages, which may lead to nondifferential bias as dietary habits are subject to alterations during the follow-up period. However, related studies of adults have pointed out that their dietary habits are not always subject to major alterations in the short term [43]. Moreover, the current evidence did not fully support the suggestion that using cumulative averages tends to produce stronger associations than using baseline dietary data [44, 45]. Second, there is a possibility of misclassification bias in the dietary data obtained from the self-reported food frequency questionnaire. Third, although these results were fully and extensively adjusted for relevant covariates, residual confounding is still difficult to completely avoid. For example, metformin use is protective against renal cancer risk in patients with diabetes [46], but it is unclear whether the diabetic population in this study was taking metformin. Fourth, the population in the study was older Americans with an average age of 65.5 years, it remains uncertain, whether the close association of DRRD with renal cancer risk can be applied to other age groups or non-U.S. populations. Future research should be performed in other populations to validate the reliability of these findings.

## Conclusion

In conclusion, this research depicted an inverse association of DRRD score with renal cancer risk. Adherence to the dietary pattern of DRRD may be an effective approach for preventing renal cancer. To enhance the reliability and scope of our findings, future research should aim to replicate our analysis in ethnically and geographically diverse cohorts, ensuring robust cross-national validation. Furthermore, conducting large-scale prospective cohort studies with extended follow-up periods will be pivotal in assessing the long-term effectiveness of adhering to the DRRD in preventing the onset and progression of renal cancer. By elucidating the generalizability and temporal sequence of this dietary pattern-disease association through rigorous longitudinal studies, we can further promote DRRD as a recommended and healthy dietary pattern for mitigating the increasing incidence of renal cancer worldwide.

### Supplementary Information

**Additional file 1:** **Table S1.** Criteria for determining diabetes risk reduction diet score. **Table S2.** Distribution of covariates with missing data before and after imputation. **Table S3.** Hazard ratios of the association of DRRD score with the risk of renal cancer in 97,486 participants with complete data. **Table S4.** Hazard ratios of the association of cereal fiber with the risk of renal cancer. ** Table S5.** Hazard ratios of the association of whole fruit intake with the risk of renal cancer. **Table S6.** Hazard ratios of the association of Sugar-sweetened beverages intake with the risk of renal cancer. **Table S7.** Hazard ratios of the association of nuts intake with the risk of renal cancer. **Table S8.** Hazard ratios of the association of coffee consumption with the risk of renal cancer. **Table S9.** Hazard ratios of the association of PUFA/SFA with the risk of renal cancer. **Table S10.** Hazard ratios of the association of Glycemic Index with the risk of renal cancer. **Table S11.** Hazard ratios of the association of TFA intake with the risk of renal cancer. **Table S12.** Hazard ratios of the association of red meat and processed meat intake with the risk of renal cancer.

## Data Availability

The raw data used in this article is not available because of the National Cancer Institute's data policy. Access to the dataset should contact the National Cancer Institute by mail.

## Abbreviations

BMI: Body mass index
BQ: Baseline questionnaire
CI: Confidence interval
DHQ: Dietary history questionnaire
HR: Hazard ratio
NCI: National cancer institute
DRRD: Diabetes risk reduction diet
PLCO: Prostate lung colorectal and ovarian

## References

[1] Scelo G, Larose TL (2018). Epidemiology and risk factors for kidney cancer. J Clin Oncol.

[2] Makino T, Kadomoto S, Izumi K, Mizokami A (2022). Epidemiology and prevention of renal cell carcinoma. Cancers (Basel).

[3] Joh HK, Willett WC, Cho E (2011). Type 2 diabetes and the risk of renal cell cancer in women. Diabetes Care.

[4] Zhang GM, Zhu Y, Ye DW (2014). Metabolic syndrome and renal cell carcinoma. World J Surg Oncol.

[5] Undzyte G, Patasius A, Linkeviciute-Ulinskiene D, Zabuliene L, Stukas R, Dulskas A (2020). Increased kidney cancer risk in diabetes mellitus patients: a population-based cohort study in Lithuania. Aging Male.

[6] Cheung CW, Vesey DA, Nicol DL, Johnson DW (2004). The roles of IGF-I and IGFBP-3 in the regulation of proximal tubule, and renal cell carcinoma cell proliferation. Kidney Int.

[7] Tracz AF, Szczylik C, Porta C, Czarnecka AM (2016). Insulin-like growth factor-1 signaling in renal cell carcinoma. BMC Cancer.

[8] Belfiore A, Malaguarnera R (2011). Insulin receptor and cancer. Endocr Relat Cancer.

[9] Rhee JJ, Mattei J, Hughes MD, Hu FB, Willett WC (2015). Dietary diabetes risk reduction score, race and ethnicity, and risk of type 2 diabetes in women. Diabetes Care.

[10] Turati F, Rossi M, Mattioli V, Bravi F, Negri E, La Vecchia C (2022). Diabetes risk reduction diet and the risk of pancreatic cancer. Eur J Nutr.

[11] Kang JH, Peng C, Rhee JJ, Farvid MS, Willett WC, Hu FB (2020). Prospective study of a diabetes risk reduction diet and the risk of breast cancer. Am J Clin Nutr.

[12] Esposito G, Bravi F, Serraino D, Parazzini F, Crispo A, Augustin LSA (2021). Diabetes risk reduction diet and endometrial cancer risk. Nutrients.

[13] Zhang Y, Zhong G, Zhu M, Chen L, Wan H, Luo F (2022). Association between diabetes risk reduction diet and lung cancer risk in 98,159 participants: results from a prospective study. Front Oncol.

[14] Prorok PC, Andriole GL, Bresalier RS, Buys SS, Chia D, Crawford ED (2000). Design of the prostate, lung, colorectal and ovarian (PLCO) cancer screening trial. Control Clin Trials.

[15] Thompson FE, Subar AF, Brown CC, Smith AF, Sharbaugh CO, Jobe JB (2002). Cognitive research enhances accuracy of food frequency questionnaire reports: results of an experimental validation study. J Am Diet Assoc.

[16] Subar AF, Thompson FE, Kipnis V, Midthune D, Hurwitz P, McNutt S (2001). Comparative validation of the block, willett, and national cancer institute food frequency questionnaires : the eating at America's table study. Am J Epidemiol.

[17] Gohagan JK, Prorok PC, Greenwald P, Kramer BS (2015). The PLCO cancer screening trial: background, goals, organization, operations, results. Rev Recent Clin Trials.

[18] Wang T, Farvid MS, Kang JH, Holmes MD, Rosner BA, Tamimi RM (2021). Diabetes risk reduction diet and survival after breast cancer diagnosis. Cancer Res.

[19] Ebrahimi Mousavi S, Bagheri A, Benisi-Kohansal S, Azadbakht L, Esmaillzadeh A (2022). Consumption of "diabetes risk reduction diet" and odds of breast cancer among women in a Middle Eastern Country. Front Nutr.

[20] Augustin LSA, Kendall CWC, Jenkins DJA, Willett WC, Astrup A, Barclay AW (2015). Glycemic index, glycemic load and glycemic response: An International Scientific Consensus Summit from the International Carbohydrate Quality Consortium (ICQC). Nutr Metab Cardiovasc Dis.

[21] Csizmadi I, Boucher BA, Lo Siou G, Massarelli I, Rondeau I, Garriguet D (2016). Using national dietary intake data to evaluate and adapt the US Diet History Questionnaire: the stepwise tailoring of an FFQ for Canadian use. Public Health Nutr.

[22] Krebs-Smith SM, Pannucci TE, Subar AF, Kirkpatrick SI, Lerman JL, Tooze JA (2018). Update of the healthy eating index: HEI-2015. J Acad Nutr Diet.

[23] Gluba-Brzózka A, Rysz J, Ławiński J, Franczyk B (2022). Renal cell cancer and obesity. Int J Mol Sci.

[24] Scelo G, Larose TL (2018). Epidemiology and risk factors for kidney cancer. J Clin Oncol.

[25] Desquilbet L, Mariotti F (2010). Dose-response analyses using restricted cubic spline functions in public health research. Stat Med.

[26] Zhao J, Zhao L (2013). Cruciferous vegetables intake is associated with lower risk of renal cell carcinoma: evidence from a meta-analysis of observational studies. PLoS ONE.

[27] Rhee J, Lim RK, Purdue MP (2022). Coffee consumption and risk of renal cancer: a meta-analysis of cohort evidence. Cancer Causes Control.

[28] Daniel CR, Cross AJ, Graubard BI, Park Y, Ward MH, Rothman N (2012). Large prospective investigation of meat intake, related mutagens, and risk of renal cell carcinoma. Am J Clin Nutr.

[29] Hu FB (2002). Dietary pattern analysis: a new direction in nutritional epidemiology. Curr Opin Lipidol.

[30] Tapsell LC, Neale EP, Satija A, Hu FB (2016). Foods, nutrients, and dietary patterns: interconnections and implications for dietary guidelines. Adv Nutr.

[31] Reedy J, Lerman JL, Krebs-Smith SM, Kirkpatrick SI, Pannucci TE, Wilson MM (2018). Evaluation of the healthy eating index-2015. J Acad Nutr Diet.

[32] Kord-Varkaneh H, Salehi-Sahlabadi A, Zarezade M, Rahmani J, Tan SC, Hekmatdoost A (2020). Association between healthy eating index-2015 and breast cancer risk: a case-control study. Asian Pac J Cancer Prev.

[33] Edefonti V, Di Maso M, Tomaino L, Parpinel M, Garavello W, Serraino D (2022). Diet quality as measured by the healthy eating index 2015 and oral and pharyngeal cancer risk. J Acad Nutr Diet.

[34] Huang TB, Ding PP, Chen JF, Yan Y, Zhang L, Liu H (2014). Dietary fiber intake and risk of renal cell carcinoma: evidence from a meta-analysis. Med Oncol.

[35] Borneo R, Leon AE (2012). Whole grain cereals: functional components and health benefits. Food Funct.

[36] Makarem N, Nicholson JM, Bandera EV, McKeown NM, Parekh N (2016). Consumption of whole grains and cereal fiber in relation to cancer risk: a systematic review of longitudinal studies. Nutr Rev.

[37] Lee JE, Giovannucci E, Smith-Warner SA, Spiegelman D, Willett WC, Curhan GC (2006). Intakes of fruits, vegetables, vitamins A, C, and E, and carotenoids and risk of renal cell cancer. Cancer Epidemiol Biomarkers Prev.

[38] Leung CY, Abe SK, Sawada N, Ishihara J, Takachi R, Yamaji T (2021). Sugary drink consumption and risk of kidney and bladder cancer in Japanese adults. Sci Rep.

[39] Guo H, Wu H, Li Z (2023). The pathogenesis of diabetes. Int J Mol Sci.

[40] Frystyk J (2004). Free insulin-like growth factors—measurements and relationships to growth hormone secretion and glucose homeostasis. Growth Horm IGF Res.

[41] Greten FR, Grivennikov SI (2019). Inflammation and cancer: triggers, mechanisms, and consequences. Immunity.

[42] Solarek W, Czarnecka AM, Escudier B, Bielecka ZF, Lian F, Szczylik C (2015). Insulin and IGFs in renal cancer risk and progression. Endocr Relat Cancer.

[43] Srour B, Fezeu LK, Kesse-Guyot E, Allès B, Méjean C, Andrianasolo RM (2019). Ultra-processed food intake and risk of cardiovascular disease: prospective cohort study (NutriNet-Santé). BMJ.

[44] Zhang J, Zhao A, Wu W, Ren Z, Yang C, Wang P (2020). Beneficial effect of dietary diversity on the risk of disability in activities of daily living in adults: a prospective cohort study. Nutrients.

[45] Hu FB, Stampfer MJ, Rimm E, Ascherio A, Rosner BA, Spiegelman D (1999). Dietary fat and coronary heart disease: a comparison of approaches for adjusting for total energy intake and modeling repeated dietary measurements. Am J Epidemiol.

[46] Tseng CH (2016). Use of metformin and risk of kidney cancer in patients with type 2 diabetes. Eur J Cancer.

