# Supplementary material for: Association of diabetes risk reduction diet with renal cancer risk in 101,755 participants: a prospective study
Source: J Transl Med. 2023 Oct 2;21:684. doi: 10.1186/s12967-023-04555-z (PMC10544595; doi:10.1186/s12967-023-04555-z)
Supplement: Supplementary file 1 — Additional file 1: Table S1. Criteria for determining diabetes risk reduction diet score. Table S2. Distribution of covariates with missing data before and after imputation. Table S3. Hazard ratios of the association of DRRD score with the risk of renal cancer in 97,486 participants with complete data. Table S4. Hazard ratios of the association of cereal fiber with the risk of renal cancer. Table S5. Hazard ratios of the association of whole fruit intake with the risk of renal cancer. Table S6. Hazard ratios of the association of Sugar-sweetened beverages intake with the risk of renal cancer. Table S7. Hazard ratios of the association of nuts intake with the risk of renal cancer. Table S8. Hazard ratios of the association of coffee consumption with the risk of renal cancer. Table S9. Hazard ratios of the association of PUFA/SFA with the risk of renal cancer. Table S10. Hazard ratios of the association of Glycemic Index with the risk of renal cancer. Table S11. Hazard ratios of the association of TFA intake with the risk of renal cancer. Table S12. Hazard ratios of the association of red meat and processed meat intake with the risk of renal cancer. [file 12967_2023_4555_MOESM1_ESM.docx]

SUPPLEMENTARY MATERIALS

**Association of Diabetes Risk Reduction Diet with Renal Cancer Risk in 101,755 Participants: A Prospective Study**

**Authors**

Ling Xiang ^1, #^, Yi Xiao ^2, #, *^, Zhiquan Xu ^2^, Haoyun Luo ^2^, Xiaorui Ren ^2^, Qi Wei ^2^, Zhiyong Zhu ^2^, Yahui Jiang ^2^, Yunhao Tang ^2^, Hongmei He ^2^, Zhihang Zhou ^3^, Haitao Gu ^2^, Yaxu Wang ^2, *^, Linglong Peng ^2, *^

**Supplementary Table 1.** Criteria for determining diabetes risk reduction diet score

| Energy-adjusted dietary intakes of individual components | | | | | | | | |  | |
| --- | --- | --- | --- | --- | --- | --- | --- | --- | --- | --- |
| Points | Cereal fiber (g/day) | Coffee  (g/day) | Nuts (g/day) | Fruit (servings/day) | Ratio of polyunsaturated to saturated fatty acids | Trans fatty acids (g/day) | Glycemic index | Red and processed meat (g/day) | | Sugar-sweetened beverages (g/day) |
| 5 | ≥15.80 | ≥1277.88 | ≥7.80 | ≥3.88 | ≥0.96 | ≤2.07 | ≤50.92 | ≤2.69 | | ≤24.88 |
| 4 | 12.17-15.79 | 1050.00-1277.87 | 3.68-7.79 | 2.72-3.87 | 0.80-0.95 | 2.08-2.97 | 50.93-52.84 | 2.70-5.50 | | 24.89-73.03 |
| 3 | 9.66-12.16 | 441.73-1050.00 | 1.40-3.67 | 1.94-2.71 | 0.68-0.79 | 2.98-3.99 | 52.85-54.39 | 5.51-9.86 | | 73.04-181.26 |
| 2 | 7.22-9.65 | 25.37-441.72 | 0.44-1.39 | 1.21-1.93 | 0.56-0.67 | 4.00-5.57 | 54.40-56.20 | 9.87-18.87 | | 181.27-389.56 |
| 1 | ≤7.21 | ≤25.36 | ≤0.43 | ≤1.20 | ≤0.55 | ≥5.58 | ≥56.21 | ≥18.88 | | ≥389.57 |

**Supplementary Table 2.** Distribution of covariates with missing data before and after imputation^*^

| Variable | Before imputation | After imputation | Number (%) with missing data |
| --- | --- | --- | --- |
| Family history of renal cancer |  |  | 781 (0.77%) |
| No | 96788 (95.9%) | 97569 (95.9%) |  |
| Yes | 1543 (1.5%) | 1543 (1.5%) |  |
| Possibly | 2643 (2.6%) | 2643 (2.6%) |  |
| Body mass index (kg/m^2^) | 27.2±4.8 | 27.2±4.8 | 1348 (1.32%) |
| Smoking status |  |  | 20 (0.02%) |
| Never | 48560 (47.7%) | 48580 (47.7%) |  |
| Current | 9401 (9.2%) | 9401 (9.2%) |  |
| Former | 43774 (43.0%) | 43774 (43.0%) |  |
| Smoking pack-years | 17.8±26.7 | 17.7±26.6 | 1164 (1.14%) |
| Ibuprofen use |  |  | 438 (0.44%) |
| No | 72911 (72.0%) | 73349 (72.1%) |  |
| Yes | 28406 (28.0%) | 28406 (27.9%) |  |
| History of hypertension |  |  | 513 (0.50%) |
| No | 68194 (67.4%) | 68707 (67.5%) |  |
| Yes | 33048 (32.6%) | 33048 (32.5%) |  |
| Weight change (pounds)^a^ | 33.0±27.2 | 33.0±27.2 | 1470(1.44%) |
| History of diabetes |  |  | 538 (0.53%) |
| No | 94411 (93.3%) | 94949 (93.3%) |  |
| Yes | 6806 (6.7%) | 6806 (6.7%) |  |

*Values are mean (standard deviation) or counts (percentage) as indicated.*

^a^ *Weight change was defined as the participant's baseline weight minus weight at age 20.*

**Supplementary Table 3.** Hazard ratios of the association of DRRD score with the risk of renal cancer in 97486 participants with complete data

| Quartiles of DRRD score | Number of cases | Person-years | Incidence rate per 1000 person-years (95% confidence interval) | Hazard ratio (95% confidence interval) | | |
| --- | --- | --- | --- | --- | --- | --- |
|  |  |  |  | Unadjusted | Model 1 ^a^ | Model 2 ^b^ |
| Quartile 1 (9-23) | 155 | 233338.2 | 0.66 (0.57, 0.78) | 1.00 (reference) | 1.00 (reference) | 1.00 (reference) |
| Quartile 2 (24-27) | 129 | 245491.2 | 0.53 (0.44, 0.62) | 0.79 (0.63, 1.00) | 0.82 (0.65, 1.04) | 0.87(0.69, 1.10) |
| Quartile 3 (28-30) | 71 | 168339.7 | 0.42 (0.33, 0.53) | 0.63 (0.48, 0.84) | 0.70 (0.52, 0.92) | 0.77 (0.58, 1.02) |
| Quartile 4 (31-45) | 77 | 215093.8 | 0.36 (0.29, 0.45) | 0.54 (0.41, 0.71) | 0.63 (0.47, 0.83) | 0.74 (0.55, 0.98) |
| P_-trend_ |  |  |  | <0.001 | <0.001 | 0.019 |

*DRRD, diabetes risk reduction diet.*

*^a^ Model 1: Adjusted for age (years), sex (male, female), race (white, non-white), marital status (married or living as married, no), educational level (college below, college graduate, postgraduate).*

*^b^ Model2: Adjusted for model 1 plus body mass index (kg/m2), smoking status (never, current, former), smoking pack-years (continuous), alcohol consumption (g/day), ibuprofen use (no, yes), arm (intervention, control), family history of renal cancer (no, yes), history of diabetes (no, yes), history of hypertension (no, yes) and energy intake from diet (kcal/day).*

| Quartiles of cereal fiber intake (g/day) | Number of participants/cases | Person-years | Incidence rate per 1000 person-years (95% confidence interval) | Hazard ratio (95% confidence interval) | | | |
| --- | --- | --- | --- | --- | --- | --- | --- |
|  |  |  |  | Unadjusted | Model 1^a^ | Model 2^b^ | |
| Quartile 1 (≤7.86) | 25480/117 | 222300.0 | 0.53 (0.44, 0.63) | 1.00 (reference) | 1.00 (reference) | | 1.00 (reference) |
| Quartile 2 (7.87-10.83) | 25403/116 | 224306.1 | 0.52 (0.43, 0.62) | 0.98 (0.76, 1.27) | 0.97 (0.75, 1.25) | | 0.93 (0.72, 1.22) |
| Quartile 3 (10.84-14.67) | 25448/110 | 226128.3 | 0.49 (0.40, 0.59) | 0.92 (0.71, 1.20) | 0.89 (0.68, 1.15) | | 0.82 (0.61, 1.10) |
| Quartile 4 (≥14.68) | 25424/103 | 226603.2 | 0.46 (0.38, 0.55) | 0.86 (0.66, 1.13) | 0.79 (0.60, 1.03) | | 0.68 (0.48, 0.97) |
| P_-trend_ |  |  |  | 0.237 | 0.060 | | 0.025 |

**Supplementary Table 4.** Hazard ratios of the association of cereal fiber with the risk of renal cancer

*^a^ Model 1: Adjusted for age (years), sex (male, female), race (white, non-white), marital status (married or living as married, no), educational level (college below, college graduate, postgraduate).*

*^b^ Model2: Adjusted for model 1 plus body mass index (kg/m2), smoking status (never, current, former), smoking pack-years (continuous), alcohol consumption (g/day), ibuprofen use (no, yes), arm (intervention, control), family history of renal cancer (no, yes), history of diabetes (no, yes), history of hypertension (no, yes) and energy intake from diet (kcal/day).*

**Supplementary Table 5.** Hazard ratios of the association of whole fruit intake with the risk of renal cancer

| Quartiles of whole fruit intake (serving/day) | Number of participants/cases | Person-years | Incidence rate per 1000 person-years (95% confidence interval) | Hazard ratio (95% confidence interval) | | | |
| --- | --- | --- | --- | --- | --- | --- | --- |
|  |  |  |  | Unadjusted | Model 1^a^ | Model 2^b^ | |
| Quartile 1 (≤1.400) | 25665/141 | 223133.6 | 0.63 (0.54, 0.76) | 1.00 (reference) | 1.00 (reference) | | 1.00 (reference) |
| Quartile 2 (1.401-2.290) | 25271/116 | 223387.7 | 0.52 (0.43, 0.62) | 0.82 (0.64, 1.05) | 0.84 (0.66, 1.08) | | 0.86 (0.67, 1.10) |
| Quartile 3 (2.291-3.510) | 25501/94 | 226838.6 | 0.41 (0.34, 0.51) | 0.66 (0.51, 0.85) | 0.69 (0.53, 0.90) | | 0.70 (0.53, 0.92) |
| Quartile 4 (≥3.511) | 25318/95 | 225977.7 | 0.42 (0.34, 0.51) | 0.67 (0.51, 0.86) | 0.70 (0.54, 0.92) | | 0.69 (0.52, 0.92) |
| P_-trend_ |  |  |  | 0.001 | 0.006 | | 0.008 |

*^a^ Model 1: Adjusted for age (years), sex (male, female), race (white, non-white), marital status (married or living as married, no), educational level (college below, college graduate, postgraduate).*

*^b^ Model2: Adjusted for model 1 plus body mass index (kg/m2), smoking status (never, current, former), smoking pack-years (continuous), alcohol consumption (g/day), ibuprofen use (no, yes), arm (intervention, control), family history of renal cancer (no, yes), history of diabetes (no, yes), history of hypertension (no, yes) and energy intake from diet (kcal/day).*

**Supplementary Table 6.** Hazard ratios of the association of Sugar-sweetened beverages intake with the risk of renal cancer

| Quartiles of Sugar-sweetened beverages intake (g/day) | Number of participants/cases | Person-years | Incidence rate per 1000 person-years (95% confidence interval) | Hazard ratio (95% confidence interval) | | | |
| --- | --- | --- | --- | --- | --- | --- | --- |
|  |  |  |  | Unadjusted | Model 1^a^ | Model 2^b^ | |
| Quartile 1 (≤ 31.95) | 25481/85 | 225288.3 | 0.38 (0.31, 0.47) | 1.00 (reference) | 1.00 (reference) | | 1.00 (reference) |
| Quartile 2 (31.96-110.22) | 25400/101 | 225925.1 | 0.45 (0.37, 0.54) | 1.18 (0.89, 1.58) | 1.14 (0.85, 1.52) | | 1.10 (0.82, 1.47) |
| Quartile 3 (110.23-318.19) | 25435/113 | 225167.1 | 0.50 (0.42, 0.60) | 1.33 (1.00, 1.76) | 1.25 (0.94, 1.67) | | 1.17 (0.88, 1.56) |
| Quartile 4 (≥ 318.19) | 25439/147 | 222957.0 | 0.66 (0.56, 0.78) | 1.75 (1.34, 2.28) | 1.68 (1.28, 2.21) | | 1.46 (1.10, 1.93) |
| P_-trend_ |  |  |  | <0.001 | <0.001 | | 0.004 |

*^a^ Model 1: Adjusted for age (years), sex (male, female), race (white, non-white), marital status (married or living as married, no), educational level (college below, college graduate, postgraduate).*

*^b^ Model2: Adjusted for model 1 plus body mass index (kg/m2), smoking status (never, current, former), smoking pack-years (continuous), alcohol consumption (g/day), ibuprofen use (no, yes), arm (intervention, control), family history of renal cancer (no, yes), history of diabetes (no, yes), history of hypertension (no, yes) and energy intake from diet (kcal/day).*

**Supplementary Table 7.** Hazard ratios of the association of nuts intake with the risk of renal cancer

| Quartiles of nuts intake (g/day) | Number of participants/cases | Person-years | Incidence rate per 1000 person-years (95% confidence interval) | Hazard ratio (95% confidence interval) | | | |
| --- | --- | --- | --- | --- | --- | --- | --- |
|  |  |  |  | Unadjusted | Model 1^a^ | Model 2^b^ | |
| Quartile 1 (≤0.48) | 25498/100 | 226343.5 | 0.44 (0.36, 0.54) | 1.00 (reference) | 1.00 (reference) | | 1.00 (reference) |
| Quartile 2 (0.49-1.79) | 29105/135 | 257683.3 | 0.52 (0.44, 0.62) | 1.19 (0.92, 1.54) | 1.01 (0.77, 1.31) | | 1.00 (0.77, 1.31) |
| Quartile 3 (1.80-7.23) | 24099/114 | 213301.7 | 0.53 (0.45, 0.64) | 1.21 (0.92, 1.58) | 1.17 (0.89, 1.53) | | 1.19 (0.91, 1.57) |
| Quartile 4 (≥7.24) | 23053/97 | 202009.1 | 0.48 (0.39, 0.59) | 1.09 (0.82, 1.44) | 0.93 (0.70, 1.23) | | 0.96 (0.71, 1.29) |
| P_-trend_ |  |  |  | 0.936 | 0.431 | | 0.589 |

*^a^ Model 1: Adjusted for age (years), sex (male, female), race (white, non-white), marital status (married or living as married, no), educational level (college below, college graduate, postgraduate).*

*^b^ Model2: Adjusted for model 1 plus body mass index (kg/m2), smoking status (never, current, former), smoking pack-years (continuous), alcohol consumption (g/day), ibuprofen use (no, yes), arm (intervention, control), family history of renal cancer (no, yes), history of diabetes (no, yes), history of hypertension (no, yes) and energy intake from diet (kcal/day).*

**Supplementary Table 8.** Hazard ratios of the association of coffee consumption with the risk of renal cancer

| Quartiles of coffee consumption (g/day) | Number of participants/cases | Person-years | Incidence rate per 1000 person-years (95% confidence interval) | Hazard ratio (95% confidence interval) | | | |
| --- | --- | --- | --- | --- | --- | --- | --- |
|  |  |  |  | Unadjusted | Model 1^a^ | Model 2^b^ | |
| Quartile 1 (≤152.1) | 25640/110 | 226287.1 | 0.49 (0.40, 0.59) | 1.00 (reference) | 1.00 (reference) | | 1.00 (reference) |
| Quartile 2 (152.2-887.1) | 26998/100 | 238684.7 | 0.42 (0.35, 0.51) | 0.86 (0.66, 1.13) | 0.84 (0.64, 1.10) | | 0.81 (0.62, 1.07) |
| Quartile 3 (887.2-1277.9) | 34279/167 | 303713.4 | 0.55 (0.47, 0.64) | 1.13 (0.89, 1.44) | 0.99 (0.78,1.26) | | 0.95 (0.74, 1.22) |
| Quartile 4 (≥1278.0) | 14838/69 | 130652.2 | 0.53 (0.42, 0.67) | 1.09 (0.80, 1.47) | 0.93 (0.69, 1.27) | | 0.86 (0.62, 1.18) |
| P_-trend_ |  |  |  | 0.206 | 0.954 | | 0.630 |

*^a^ Model 1: Adjusted for age (years), sex (male, female), race (white, non-white), marital status (married or living as married, no), educational level (college below, college graduate, postgraduate).*

*^b^ Model2: Adjusted for model 1 plus body mass index (kg/m2), smoking status (never, current, former), smoking pack-years (continuous), alcohol consumption (g/day), ibuprofen use (no, yes), arm (intervention, control), family history of renal cancer (no, yes), history of diabetes (no, yes), history of hypertension (no, yes) and energy intake from diet (kcal/day).*

**Supplementary Table 9.** Hazard ratios of the association of PUFA/SFA with the risk of renal cancer

| Quartiles of PUFA/SFA (g/day) | Number of participants/cases | Person-years | Incidence rate per 1000 person-years (95% confidence interval) | Hazard ratio (95% confidence interval) | | | |
| --- | --- | --- | --- | --- | --- | --- | --- |
|  |  |  |  | Unadjusted | Model 1^a^ | Model 2^b^ | |
| Quartile 1 (≤ 0.586) | 25439/130 | 221842.4 | 0.59 (0.49, 0.70) | 1.00 (reference) | 1.00 (reference) | | 1.00 (reference) |
| Quartile 2 (0.587-0.727) | 25439/108 | 224847.6 | 0.48 (0.40, 0.58) | 0.82 (0.63, 1.06) | 0.88 (0.68, 1.13) | | 0.89 (0.69, 1.15) |
| Quartile 3 (0.728-0.896) | 25438/100 | 225644.4 | 0.44 (0.36, 0.54) | 0.76 (0.58, 0.98) | 0.86 (0.66, 1.12) | | 0.89 (0.68, 1.16) |
| Quartile 4 (≥0.897) | 25439/108 | 227003.2 | 0.48 (0.39, 0.57) | 0.81 (0.63, 1.05) | 0.99 (0.76, 1.29) | | 1.07 (0.82, 1.40) |
| P_-trend_ |  |  |  | 0.115 | 0.964 | | 0.590 |

*PUFA, polyunsaturated fatty acids; SFA, saturated fatty acids.*

*^a^ Model 1: Adjusted for age (years), sex (male, female), race (white, non-white), marital status (married or living as married, no), educational level (college below, college graduate, postgraduate).*

*^b^ Model2: Adjusted for model 1 plus body mass index (kg/m2), smoking status (never, current, former), smoking pack-years (continuous), alcohol consumption (g/day), ibuprofen use (no, yes), arm (intervention, control), family history of renal cancer (no, yes), history of diabetes (no, yes), history of hypertension (no, yes) and energy intake from diet (kcal/day).*

| Quartiles of Glycemic Index | Number of participants/cases | Person-years | Incidence rate per 1000 person-years (95% confidence interval) | Hazard ratio (95% confidence interval) | | | |
| --- | --- | --- | --- | --- | --- | --- | --- |
|  |  |  |  | Unadjusted | Model 1^a^ | Model 2^b^ | |
| Quartile 1 (≤51.48) | 25464/91 | 223584.5 | 0.41 (0.33, 0.50) | 1.00 (reference) | 1.00 (reference) | | 1.00 (reference) |
| Quartile 2 (51.49-53.62) | 25488/90 | 225495.6 | 0.40 (0.33, 0.49) | 0.98 (0.73, 1.31) | 0.94 (0.70, 1.26) | | 0.92 (0.689, 1.24) |
| Quartile 3 (53.63-55.68) | 25388/135 | 225297.7 | 0.60 (0.51, 0.71) | 1.47 (1.13, 1.92) | 1.37 (1.05, 1.79) | | 1.32 (1.008, 1.72) |
| Quartile 4 (≥55.69) | 25415/130 | 224959.8 | 0.58 (0.49, 0.69) | 1.42 (1.09, 1.86) | 1.29 (0.99, 1.69) | | 1.22 (0.93, 1.60) |
| P_-trend_ |  |  |  | 0.001 | 0.012 | | 0.042 |

**Supplementary Table 10.** Hazard ratios of the association of Glycemic Index with the risk of renal cancer

*^a^ Model 1: Adjusted for age (years), sex (male, female), race (white, non-white), marital status (married or living as married, no), educational level (college below, college graduate, postgraduate).*

*^b^ Model2: Adjusted for model 1 plus body mass index (kg/m2), smoking status (never, current, former), smoking pack-years (continuous), alcohol consumption (g/day), ibuprofen use (no, yes), arm (intervention, control), family history of renal cancer (no, yes), history of diabetes (no, yes), history of hypertension (no, yes) and energy intake from diet (kcal/day).*

| Quartiles of TFA intake (g/day) | Number of participants/cases | Person-years | Incidence rate per 1000 person-years (95% confidence interval) | Hazard ratio (95% confidence interval) | | | |
| --- | --- | --- | --- | --- | --- | --- | --- |
|  |  |  |  | Unadjusted | Model 1^a^ | Model 2^b^ | |
| Quartile 1 (≤2.30) | 25659/94 | 227636.1 | 0.41 (0.34, 0.51) | 1.00 (reference) | 1.00 (reference) | | 1.00 (reference) |
| Quartile 2 (2.31-3.45) | 25384/102 | 224871.1 | 0.45 (0.37, 0.55) | 1.10 (0.83, 1.45) | 1.03 (0.78, 1.37) | | 1.01 (0.76, 1.35) |
| Quartile 3 (3.46-5.08) | 25354/104 | 224416.7 | 0.46 (0.38, 0.56) | 1.12 (0.85, 1.48) | 0.98 (0.74, 1.30) | | 0.95 (0.70, 1.30) |
| Quartile 4 (≥5.09) | 25358/146 | 222413.5 | 0.66 (0.56, 0.77) | 1.59 (1.23, 2.06) | 1.24 (0.94, 1.63) | | 1.19 (0.81, 1.73) |
| P_-trend_ |  |  |  | <0.001 | 0.087 | | 0.332 |

**Supplementary Table 11.** Hazard ratios of the association of TFA intake with the risk of renal cancer

*TFA, trans fatty acids.*

*^a^ Model 1: Adjusted for age (years), sex (male, female), race (white, non-white), marital status (married or living as married, no), educational level (college below, college graduate, postgraduate).*

*^b^ Model2: Adjusted for model 1 plus body mass index (kg/m2), smoking status (never, current, former), smoking pack-years (continuous), alcohol consumption (g/day), ibuprofen use (no, yes), arm (intervention, control), family history of renal cancer (no, yes), history of diabetes (no, yes), history of hypertension (no, yes) and energy intake from diet (kcal/day).*

**Supplementary Table 12.** Hazard ratios of the association of red meat and processed meat intake with the risk of renal cancer

| Quartiles of red meat and processed meat intake (g/day) | Number of participants/cases | Person-years | Incidence rate per 1000 person-years (95% confidence interval) | Hazard ratio (95% confidence interval) | | | |
| --- | --- | --- | --- | --- | --- | --- | --- |
|  |  |  |  | Unadjusted | Model 1^a^ | Model 2^b^ | |
| Quartile 1 (≤ 3.32) | 25462/76 | 227589.2 | 0.33 (0.27, 0.42) | 1.00 (reference) | 1.00 (reference) | | 1.00 (reference) |
| Quartile 2 (3.33-7.41) | 25444/92 | 225752.6 | 0.41 (0.33, 0.50) | 1.22 (0.90, 1.65) | 1.12 (0.83, 1.52) | | 1.05 (0.78, 1.43) |
| Quartile 3 (7.42-15.79) | 25425/136 | 223945.7 | 0.61 (0.51, 0.72) | 1.82 (1.37, 2.41) | 1.53 (1.15, 2.04) | | 1.38 (1.03, 1.86) |
| Quartile 4 (≥15.80) | 25424/142 | 222049.9 | 0.64 (0.54, 0.75) | 1.92 (1.45, 2.53) | 1.44 (1.08, 1.94) | | 1.26 (0.92, 1.73) |
| P_-trend_ |  |  |  | <0.001 | 0.024 | | 0.231 |

*^a^ Model 1: Adjusted for age (years), sex (male, female), race (white, non-white), marital status (married or living as married, no), educational level (college below, college graduate, postgraduate).*

*^b^ Model2: Adjusted for model 1 plus body mass index (kg/m2), smoking status (never, current, former), smoking pack-years (continuous), alcohol consumption (g/day), ibuprofen use (no, yes), arm (intervention, control), family history of renal cancer (no, yes), history of diabetes (no, yes), history of hypertension (no, yes) and energy intake from diet (kcal/day).*
